# Binocular Vision-Based Image Extraction and Feature Analysis of Weld Beads in 316L Wire Arc Additive Manufacturing

**DOI:** 10.3390/mi17070860

**Published:** 2026-07-20

**Authors:** Youshu Yue, Qiang Zhu, Huan Li

**Affiliations:** 1 School of Robotics, Guangdong Open University, Guangzhou 510091, China; ysle@gdpi.edu.cn (Y.Y.); qzhu@gdpi.edu.cn (Q.Z.); 2 School of Mechanical Engineering, Yangtze University, Jingzhou 519016, China

**Keywords:** arc additive manufacturing, dynamic tracking, image processing, weld seam pixel area, molten pool pixel area

## Abstract

To address the challenges of low image quality and difficult feature extraction of weld beads caused by the complex dynamics of the molten pool, intense arc light, and spatter interference during wire arc additive manufacturing (WAAM) of 316L stainless steel, this paper develops a binocular vision-based dynamic molten pool tracking system and conducts image processing and feature analysis. Two high-speed CMOS cameras are employed to capture images of the molten pool and weld bead. Camera calibration is performed to convert pixel coordinates to world coordinates. The denoising performance of five filtering methods, namely mean, Gaussian, median, maximum, and minimum filters, is systematically compared, and the minimum filter is selected for noise reduction. Adaptive threshold binarization, adapthisteq image enhancement, and morphological threshold segmentation are integrated to effectively separate the weld bead from the background. Four edge detection algorithms—Sobel, Robert, Laplacian, and Canny—are compared, and the Canny algorithm combined with Hough transform line fitting is determined to achieve complete and continuous extraction of the weld bead contour. The Intersection over Union (IoU) metric is introduced for image quality screening. When IoU is set to 0.3, the detection accuracy exceeds 90%, effectively eliminating defective images caused by spatter, explosion, trailing, and other disturbances. The proposed method facilitates stable extraction of geometric parameters (e.g., pixel area of the weld bead and height/width of the molten pool), thereby offering a feasible image-processing solution for dynamic molten-pool monitoring and online quality assessment of 316L stainless steel components fabricated by wire arc additive manufacturing.

## 1. Introduction

316L stainless steel structural components fabricated via wire arc additive manufacturing (WAAM) have been successfully applied in aerospace, petroleum engineering, and related fields, owing to their outstanding mechanical properties and forming efficiency [1,2,3]. This technology relies on high-performance trajectory planning strategies to enable near-net-shape fabrication of geometrically complex components and is expected to extend its applications to a broader range of industrial sectors in the future. Nevertheless, current quality assessment of metallic WAAM parts primarily relies on post-deposition destructive mechanical testing, which is inadequate for in-process evaluation [4,5]. Previous studies have demonstrated that the forming quality of additive manufacturing components is closely correlated with weld bead geometry [6,7,8]. Since molten pool tracking combined with image processing techniques enables real-time detection of bead morphology, this technological approach holds significant potential for online quality assessment in WAAM processes [9,10,11].

Dynamic molten pool tracking serves as a core element in ensuring the manufacturing quality and geometric precision of additive manufacturing [4,12,13]. Its primary function is to enable precise control over the molten pool behavior through real-time acquisition of dynamic characteristics, including pool morphology, dimensions, temperature distribution, and fluid flow status, thereby ensuring the geometric accuracy, surface integrity, and microstructural homogeneity of the final components [14,15,16]. The associated image processing techniques rely on high-resolution imaging systems to capture dynamic images of the molten pool and employ advanced algorithms to extract critical feature parameters [17]. In recent years, extensive research has been conducted on molten pool image analysis during welding and additive manufacturing processes. Xiong et al. [18] designed a passive visual sensing system consisting of an industrial camera and a biprism. Through a series of procedures, including camera calibration, image correction, molten pool extraction, and edge reconstruction, they achieved real-time layer-width extraction. Their findings revealed that passive visual perception combined with a feedback control strategy significantly improved layer-width stability and that this stability increased with the number of deposited layers. After reaching a certain layer count, the maximum width deviation between adjacent weld beads was reduced to less than 0.5 mm. Jiao et al. [11] developed a visual acquisition system using a high-speed CCD camera and employed the Sobel edge detection operator to extract pool edge contours, effectively mitigating interference from arc light on imaging quality. Feng et al. [19] proposed a semi-automatic video annotation tool for WAAM processes based on the Segment Anything Model (SAM) and the XMem video object tracking model. This tool annotates video frames at a speed approximately two orders of magnitude faster than conventional manual labeling, enabling rapid quantitative analysis of the additive manufacturing process. Its dynamic tracking stability and reliability were validated through online bead-width closed-loop control, droplet transfer behavior analysis, and the construction of specialized deep-learning segmentation datasets. Liu et al. [8] introduced a molten pool edge detection method that integrates dark channel prior dehazing (DCPD) with an improved single-scale Retinex image enhancement algorithm. This approach effectively addresses the issues of excessive edge noise in raw pool images and the difficulty in feature extraction from dark regions after DCPD processing. Comparative experiments and ablation studies demonstrated that the proposed algorithm substantially enhances both image enhancement performance and feature extraction accuracy, enabling accurate and complete reconstruction of the molten pool contour. However, studies focusing on image processing for WAAM of 316L stainless steel are still scarce, thereby presenting obstacles to the advancement of high-quality additive manufacturing of this material.

Therefore, in this study, real-time image extraction and processing of weld bead and molten pool morphology during wire arc additive manufacturing of 316L stainless steel were carried out. The acquired real-time images were decomposed frame by frame, and a series of algorithms, including image enhancement, noise reduction, grayscale binarization, and morphological threshold segmentation, were employed to separate the molten pool from the background. In addition, the extraction performance of multiple edge detection algorithms was compared. The findings of this work provide guidance for further implementation of intelligent quality monitoring in wire arc additive manufacturing of 316L stainless steel.

## 2. Materials and Methods

### 2.1. WAAM Experimental Setup

**Figure 1:**
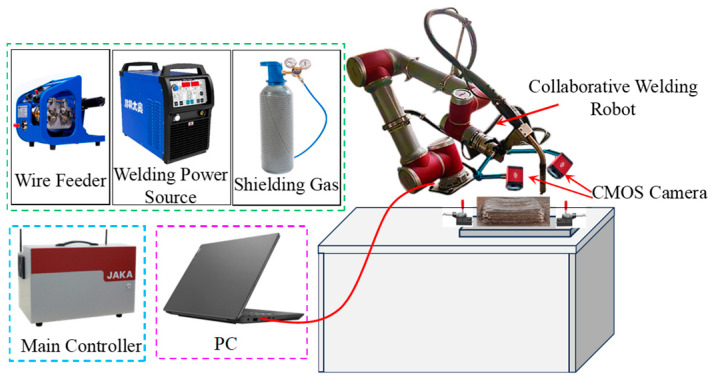
Schematic of the wire arc additive manufacturing and image acquisition system.

**Figure 2:**
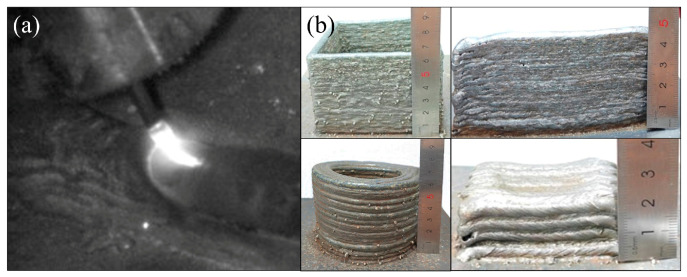
Arc morphology acquisition samples and printed specimens of 316L stainless steel fabricated by wire arc additive manufacturing. (**a**) Examples of collected arc morphology; (**b**) 316L stainless steel wire specimens.

The overall configuration of the wire arc additive manufacturing (WAAM) system, along with the weld bead and molten pool tracking subsystems, is illustrated in Figure 1. The system primarily comprises the motion execution unit—i.e., the welding robot body—along with a welding power source, a main control cabinet, a wire feeder, a shielding gas supply unit, and a worktable. The welding robot is a JAKA Zu12 from Shanghai JAKA Robotics Technology Co., Ltd., China, collaborative robot with six degrees of freedom, featuring a rated payload of 12 kg, a maximum working radius of 1327 mm, and a repeated positioning accuracy of 0.03 mm. The end-effector, equipped with a welding torch, enables high-precision wire arc additive manufacturing. The main control cabinet, model JAKA CAB12, supports both Modbus TCP/IP and Modbus RTU communication protocols, while the welding robot is compatible with the Profinet protocol, allowing reliable data exchange with the control cabinet. The welding power source is a NBC-350RL model from Shandong Aotai Electric Co., Ltd., China, operating in a constant-voltage control mode and incorporating a dedicated stainless steel welding process mode. The welding power source operates in a pulsed gas metal arc welding (GMAW) mode with a synergic control, and the metal transfer is of the short-circuiting type, which is common for 316L stainless steel. The weld bead and molten pool dynamic tracking system consists of two CMOS cameras and a personal computer. Figure 2 presents typical WAAM-fabricated 316L stainless steel specimens, as well as representative samples used for arc and weld bead image acquisition.

The nominal welding current and voltage are set at 180 A and 22 V, respectively, with a travel speed of 300 mm/min and a wire feed speed of 6.5 m/min. A filler wire of ER316L stainless steel with a diameter of 1.2 mm was selected, and its chemical composition is presented in Table 1, which is closely comparable to that of 316L stainless steel. The substrate material was a Q235 steel plate with dimensions of 200 mm × 200 mm × 10 mm. A shielding gas mixture of 95% Ar + 5% CO_2_ was employed at a flow rate of 24 L/min. The deposition followed a multi-layer single-pass strategy with a unidirectional raster path (alternating direction for each layer) and a 50% overlap ratio between adjacent beads. To prevent an excessively high temperature gradient between the substrate and the deposited layers, which could induce thermal stress and deformation, the substrate was preheated prior to deposition. Furthermore, based on multiple experimental trials, an interlayer idle time of 3 min was determined to be effective in maintaining the interpass temperature within the range of 120 °C to 140 °C. This temperature range is favorable for exerting a tempering effect, thereby enhancing both the microstructure and mechanical properties of the WAAM-fabricated components.

### 2.2. Camera Calibration and Coordinate System Transformation for Arc Tracking and Monitoring

**Figure 3:**
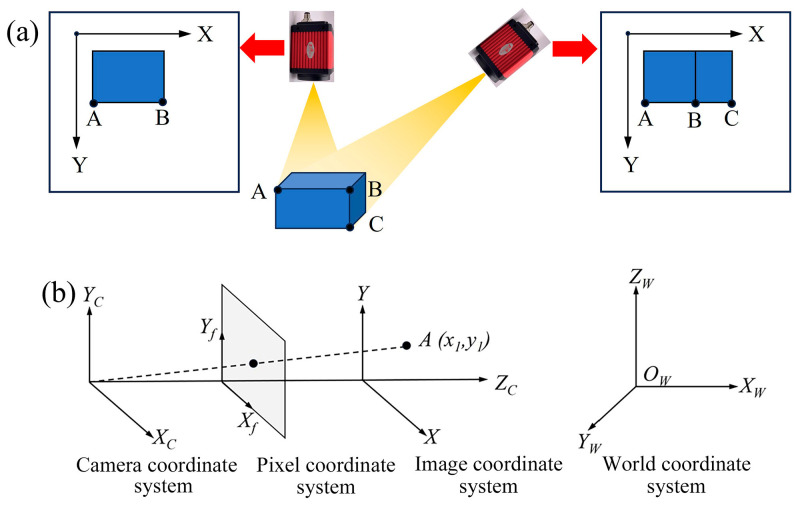
(**a**) Camera calibration principle and (**b**) camera coordinate system transformation.

Since the molten pool images captured by the CMOS camera are two-dimensional representations that cannot directly reflect the true three-dimensional morphological characteristics of the pool, camera calibration is required (as shown in Figure 3a). The objective of this calibration is to convert the pixel dimensions of the 2D images into three-dimensional spatial geometric dimensions, thereby eliminating the distortion effects of lens aberration on the molten pool morphology and enabling spatiotemporally refined analysis of the dynamic pool behavior. In this experiment, the CMOS camera was rigidly mounted on the welding torch and moved with the robotic arm; therefore, an active camera calibration method was adopted to obtain both the intrinsic and extrinsic parameters of the camera. To facilitate the determination of these parameters, the entire calibration system was divided into four coordinate systems based on the pinhole imaging principle, namely the world coordinate system, the camera coordinate system, the image coordinate system, and the pixel coordinate system, as illustrated in Figure 3b.

The transformation relationship between the world coordinate system and the pixel coordinate system is given as follows [20]:

(1) $$\left[\begin{array}{l} u\\ v\\ 1 \end{array}\right]=\frac{1}{Z_{C}}\left[\begin{array}{cccc} \alpha & 0 & u_{0} & 0\\ 0 & \beta & v_{0} & 0\\ 0 & 0 & 1 & 0 \end{array}\right]\times \left[\begin{array}{cc} R & \tau \\ 0 & 1 \end{array}\right]\times \left[\begin{array}{c} X_{W}\\ Y_{W}\\ Z_{W}\\ 1 \end{array}\right]$$

The computation formulas for the camera intrinsic parameter matrix *M* and the extrinsic parameter matrix *N* are given as follows [20]:

(2) $$\left\{\begin{array}{l} M=\left[\begin{array}{cccc} \alpha & 0 & u_{0} & 0\\ 0 & \beta & v_{0} & 0\\ 0 & 0 & 1 & 0 \end{array}\right]\\ N=\left[\begin{array}{cc} R & \tau \\ 0 & 1 \end{array}\right] \end{array}\right.$$

where *α* = *f*/*dx* and *β* = *f*/*dy*, in which f denotes the distance from the camera optical center to the imaging plane (i.e., the focal length), and *dx* and *dy* represent the physical dimensions corresponding to a unit pixel along the *x*-axis and *y*-axis directions of the image plane, respectively; *Z_C_* is the depth coordinate (i.e., the longitudinal coordinate) of a spatial point in the camera coordinate system; u and v are the horizontal and vertical coordinates in the image pixel coordinate *system*, respectively; u0 and v0 denote the coordinates of the intersection point between the actual image plane and the camera optical axis, i.e., the principal point coordinates; *Xw*, *Yw*, and *Zw* are the horizontal, vertical, and elevational coordinates of a spatial point in the world coordinate system, respectively; *R* is the rotation matrix from the camera coordinate system to the world coordinate system, and *τ* is the translation vector (i.e., the three-dimensional positional coordinates) of the origin of the camera coordinate system in the world coordinate system.

**Figure 4:**
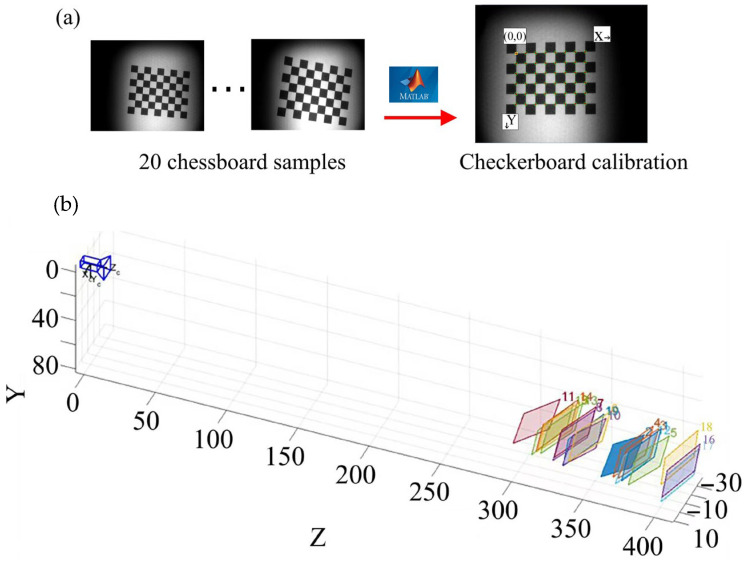
(**a**) MATLAB-based camera chessboard calibration and (**b**) the corresponding calibration principle.

However, the above imaging principle is only valid under ideal conditions. In the actual wire arc additive manufacturing process, the camera optical axis tends to deviate from the image plane, which disrupts the collinearity condition (i.e., the “three points on a line” constraint) of the light rays. Therefore, during dynamic molten pool tracking, it is necessary to analyze and correct the imaging distortions in both the *x* and *y* directions based on the actual deviations. The corresponding formulas are given as follows [21]:

(3) $$\left\{\begin{array}{l} x_{u}=x_{d}+\sigma_{x\left(x,y\right)}\\ y_{u}=y_{d}+\sigma_{y\left(x,y\right)} \end{array}\right.$$

where *σ_x_*(*x*, *y*) and *σ_γ_*(*x*, *y*) denote the distortion values in the *x* and *y* directions, respectively; *x_d_* and *y_d_* are the actual image coordinates in the *x* and *y* directions, respectively; and *x_u_* and *y_u_* are the ideal undistorted coordinate values in the *x* and *y* directions, corresponding to the radial distortion terms, respectively. Among various distortion sources, radial distortion is the predominant factor contributing to errors in camera imaging systems, and can be categorized into positive and negative radial distortion. Its mathematical formulation is given as follows:

(4) $$\left\{\begin{array}{l} \sigma_{x(x,y)}=x\left[k_{1}(x^{2}+y^{2})+k_{2}{\left(x^{2}+y^{2}\right)}^{2}\right]\\ \sigma_{y(x,y)}=y\left[k_{1}(x^{2}+y^{2})+k_{2}{\left(x^{2}+y^{2}\right)}^{2}\right] \end{array}\right.$$

where *k_1_* and *k_2_* are the first-order and second-order radial distortion coefficients, respectively. Camera calibration was performed using MATLAB R2024a. A checkerboard pattern with 9 × 7 internal corners was adopted as the calibration target, with each individual square having an actual side length of 5 mm. A total of 20 calibration images were collected as samples. The calibration procedure was carried out using the Camera Visualizer module in MATLAB, as shown in Figure 4, and the resulting camera parameters are listed in Table 2.

The calibrated binocular setup enables stereo matching and depth recovery from disparity, offering the potential for three-dimensional surface reconstruction. However, since the cameras cannot penetrate the molten pool and the present study prioritizes robust 2D contour extraction under severe interferences, we focus on planar feature parameters, namely area, width, and height.

### 2.3. Image Processing, Feature Region Extraction, and Calculation

Real-time monitoring of molten pool images enables direct assessment of the cladding status during the deposition process. In wire arc additive manufacturing, a more stable molten pool morphology generally contributes to higher geometric accuracy of the fabricated components. However, under actual processing conditions, image quality is susceptible to various interfering factors, including welding fume, spatter particles, and intense arc light [18]. Therefore, to effectively identify the weld bead and accurately extract its features, it is essential to perform noise reduction on the acquired images, aiming to suppress noise levels and enhance image quality and clarity. Currently, the commonly employed image denoising methods primarily fall into two categories: spatial-domain filtering and frequency-domain filtering. In this study, spatial-domain filtering is adopted, which operates directly within the pixel space by modifying pixel values and their neighborhood values to achieve the filtering effect. Equation (5) presents the mathematical formulation of spatial-domain filtering, whose underlying principle involves performing a convolution operation to compute the weighted sum of the filter kernel with the local region of the image, thereby enabling image smoothing or enhancement.

(5) $$g(x,y)=\sum_{i=-a}^{a}\sum_{j=-b}^{b}f(x+i,y+j)\times \omega (i,j)$$

where *g*(*x*, *y*) and *f*(*x*, *y*) denote the output and input images, respectively, and *ω*(*i*, *j*) represents the filter kernel. It can be seen from this formulation that the core of image noise reduction lies in the selection of the filter kernel. To this end, both linear filtering (including mean filter and Gaussian filter) and nonlinear filtering (including median filter as well as maximum and minimum filters) were employed in this study for noise reduction in the weld bead images.

In addition, weld bead images are susceptible to interference from factors such as fume and insufficient illumination, which may cause loss of image details and consequently hinder the accurate extraction of weld feature regions. Therefore, image enhancement of the weld bead images is required. The preprocessing procedure is as follows: the original weld images are first converted to grayscale, followed by noise reduction using median filtering (for salt-and-pepper noise removal) and Gaussian filtering (for image smoothing). Subsequently, the adapthisteq function is applied to perform adaptive histogram equalization, thereby enhancing image contrast, sharpening critical weld details, and highlighting the weld contour. During this process, the grayscale levels must be strictly maintained within the range of 0 to 255 to prevent overflow. Since the binarized weld images may still contain substantial speckle noise, non-weld regions (such as background interference and incomplete target areas) are often misclassified as weld features, interfering with the identification and extraction of the weld feature regions. Morphological threshold segmentation operates on binary grayscale images at the pixel level based on structuring elements, with core operations including erosion, dilation, opening, and closing. Consequently, morphological threshold segmentation is required to eliminate interfering regions while preserving the true weld area. Based on the binarized weld images, the screening criteria for weld morphology are established as follows: the pixel area of the weld must be greater than or equal to the minimum acceptable pixel area, the circularity must be no less than the minimum circularity threshold, and the eccentricity must be no greater than the maximum eccentricity threshold.

After morphological threshold segmentation, only the critical weld region remains in the image. To extract edge information features of the weld for further analysis of its geometry, dimensions, and dynamic behavior and to compare these with actual measurements for evaluating the accuracy (mean error and precision) of the dynamic monitoring system in feature size determination—thereby achieving real-time trajectory tracking of the weld, it is necessary to perform edge feature segmentation and extraction on the weld. Edge feature extraction facilitates the assessment of whether the weld morphology is regular and whether defects such as cracks or porosity are present. Currently, commonly employed edge detection algorithms include first-order and second-order edge detection operators. First-order operators include the Sobel operator and the Robert operator [19], while second-order operators include the Laplacian operator and the Canny operator. In this study, all four aforementioned algorithms were selected for weld contour extraction. The computation of weld feature regions primarily involves the determination of parameters such as weld pixel area, molten pool height, and width. The general processing workflow in current research is as follows: first, image preprocessing is performed for noise reduction to eliminate interfering factors; second, morphological threshold segmentation is applied to separate weld features from the background; finally, the pixel counting method is employed to tally all pixels in the image, and in conjunction with the camera calibration parameters, the conversion relationship between pixel units and physical dimensions is established, thereby yielding the actual weld feature values.

In summary, although the molten pool and the weld bead are captured within the same experimental setup, they are processed with distinct image analysis strategies due to their inherent physical differences. The molten pool appears as a highly dynamic and intensely illuminated region with rapidly varying boundaries, which requires more aggressive contrast enhancement and adaptive thresholding to reliably extract its transient contour. In contrast, the weld bead represents the solidified morphology and is primarily contaminated by spatter particles and fume, thus demanding rigorous morphological filtering and precise edge detection, such as the Canny operator combined with Hough transform, to obtain clean and continuous outlines. Despite these divergent treatments, both pipelines share a common foundation, including grayscale conversion, minimum filtering for noise suppression, and binarization, while the specific parameter sets and subsequent operations are tailored to each target. Moreover, the two processing streams are not independent; the weld bead is derived from the same welding pass as the molten pool, and the geometric features extracted from both are interrelated through the deposition dynamics and camera calibration. Ultimately, the integration of these complementary processing strategies enables a comprehensive characterization of the forming process, laying a solid basis for subsequent quantitative analysis and online quality assessment.

### 2.4. Geometric Morphology Analysis of Arc and Weld Bead

**Figure 5:**
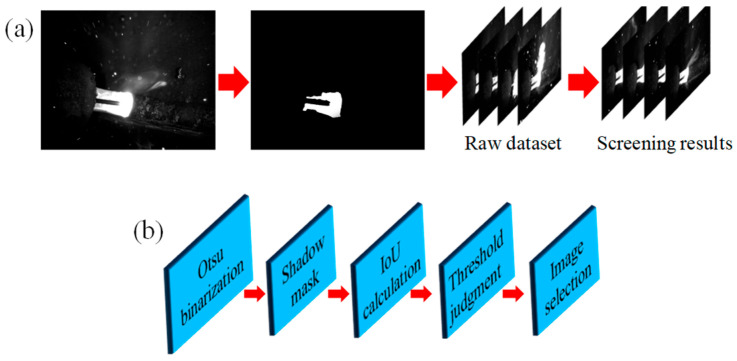
(**a**) Screening criteria for molten pool images; (**b**) Extraction procedure of molten pool morphology.

**Figure 6:**
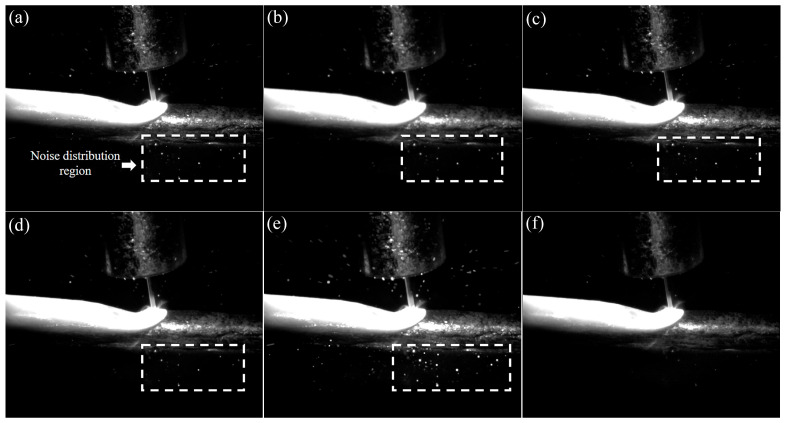
Weld morphology obtained using different filters. (**a**) Original image; (**b**) Gaussian filtering; (**c**) Mean filtering; (**d**) Median filtering; (**e**) Maximum filtering; (**f**) Minimum filtering.

Considering that frame-by-frame decomposition of video generates a substantial number of images, and not all decomposed frames can clearly present the morphological features of the molten pool and weld bead with sufficient analytical value, including all frames in subsequent processing would significantly increase computational load and reduce research efficiency. To address this issue, a targeted image screening criterion was established in this study, with the procedure illustrated in Figure 5a. First, based on key quality indicators such as molten pool fullness and weld bead regularity, a representative image exhibiting favorable weld morphology and minimal interference was selected from the decomposed frames as the standard reference image (Figure 6a). This reference image was then subjected to a series of preprocessing operations, including image enhancement, grayscale binarization, and morphological threshold segmentation, to optimize image quality and highlight weld features. Subsequently, the Otsu threshold value (Otsu value) of the processed reference image was computed. Meanwhile, the pixel values in the dark regions of the image were uniformly set to zero, and a dark-region mask was applied to mitigate interference from dark areas in subsequent similarity comparison. Subsequently, the Intersection over Union (IoU) metric was adopted as the similarity evaluation index to compare each decomposed original image against the standard reference image in a sequential manner. Images exhibiting high similarity to the reference standard were designated as valid screened images, whereas those affected by interfering factors such as spatter, arc explosion, or fume occlusion that prevented clear identification of molten pool and weld morphology were uniformly classified as interfering images. It should be particularly noted that if an interfering image is erroneously classified as a valid screened image, such an event is defined as a misjudgment in this study. Subsequent optimization of the screening threshold strategy will be conducted to reduce the misjudgment probability, thereby ensuring the reliability of the screened image dataset. Given that the key to image screening lies in the IoU value of the dark-region masks between two images, a welding video segment was randomly selected in this study. Based on the 397 molten pool images from the original dataset, image screening experiments were conducted under different IoU threshold conditions to evaluate the effect of threshold settings on screening performance. Figure 5b and Figure 6b present a schematic diagram of the weld morphology extraction procedure.

## 3. Results and Discussion

### 3.1. Results of Image Denoising and Image Enhancement

Figure 6 presents the denoising results of weld bead images obtained using different filtering methods. Figure 6a shows the original extracted weld image, in which spatter particles generated during the wire arc additive manufacturing process appear as distinct bright spots, potentially interfering with the subsequent accurate extraction of weld edge contours. To suppress the aforementioned noise, Gaussian filtering (Figure 6b) and mean filtering (Figure 6c) were first applied. Both methods smooth the overall grayscale distribution of the image through weighted averaging of pixel neighborhoods; however, they fail to effectively eliminate the bright spots caused by spatter particles. In comparison, median filtering (Figure 6d), which replaces the central pixel value with the median of its neighboring pixels, yields slightly better denoising performance than Gaussian and mean filtering, yet still cannot completely remove the interference of spatter particles from the weld images. When maximum filtering was further employed (Figure 6e), although this method effectively removes dark spots and preserves detailed features in bright regions, it paradoxically exacerbates the spatter-induced interference. In contrast, when minimum filtering was applied (Figure 6f), the majority of bright spot noise was eliminated, with no white speckles remaining in the weld image, while the primary structural details of the image were well preserved without over-processing. Based on a comprehensive comparison of the above methods, minimum filtering was ultimately selected for noise reduction in the weld bead images in this study.

### 3.2. Grayscale Image Binarization

Grayscale binarization of weld bead images is a critical step to ensure accurate extraction of weld edge contours. Its purpose is to simplify image information and eliminate color interference, thereby highlighting the target features of the weld. Specifically, pixels with grayscale values T satisfying 240 ≤ T ≤ 255 are assigned white (pixel value = 255), while all other pixels are assigned black (pixel value 0). This lays the foundation for subsequent quantitative analysis of weld feature regions. Insufficient binarization would leave residual interference in the image, leading to errors in feature extraction.

In this study, an adaptive threshold binarization method was employed, with relevant parameters listed in Table 3. This method dynamically adjusts the threshold based on the grayscale distribution of local image regions. The image is divided into multiple small subregions, and an optimal threshold is independently computed for each subregion, thereby effectively avoiding the loss of local information that commonly occurs with traditional global threshold binarization.

**Figure 7:**
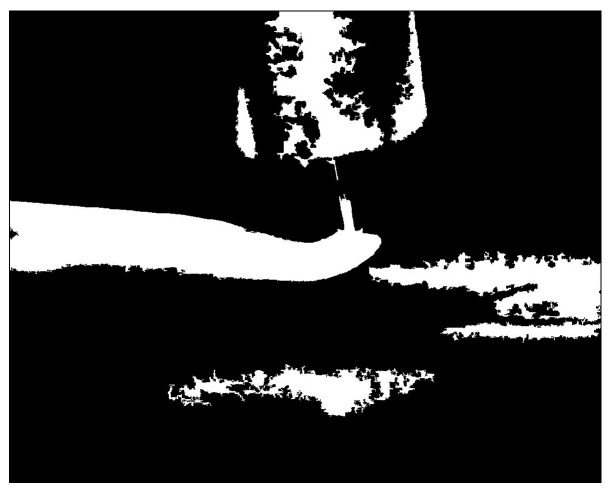
Weld image after grayscale binarization.

Figure 7 presents the result of the weld image after grayscale binarization. It can be observed that the processed image retains only black-and-white binary regions, with the weld target region successfully separated from the background, and the vast majority of non-weld feature regions have been eliminated.

### 3.3. Weld Image Enhancement Results

**Figure 8:**
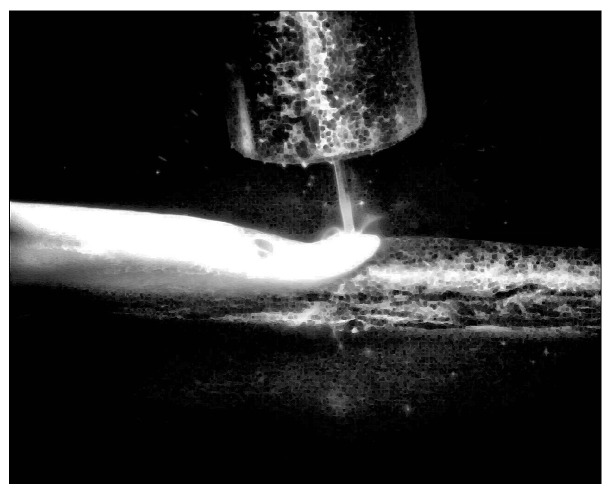
Weld image after enhancement processing.

Weld bead images are susceptible to interference from factors such as fume and insufficient illumination, which may cause loss of image details and consequently hinder the accurate extraction of weld feature regions. Therefore, image enhancement of the weld images is required, with the results presented in Figure 8. The specific preprocessing procedure is as follows: the original weld images are first converted to grayscale, followed by sequential noise reduction using median filtering (for salt-and-pepper noise removal) and Gaussian filtering (for image smoothing). On this basis, the adapthisteq function is employed to perform adaptive histogram equalization, thereby enhancing image contrast, sharpening critical weld details, and highlighting the weld contour. It should be particularly noted that the pixel grayscale values must be strictly maintained within the range of 0 to 255 throughout the process to prevent overflow.

### 3.4. Image Feature Extraction Results

#### 3.4.1. Morphological Threshold Segmentation of Weld Morphology

After binarization, weld bead images may still contain large-area speckle noise, and non-weld regions (such as background interference and incomplete target areas) are often misclassified as weld features, interfering with the accurate identification and extraction of weld feature regions. Therefore, morphological threshold segmentation is required to eliminate interfering regions while preserving the true weld area.

**Figure 9:**
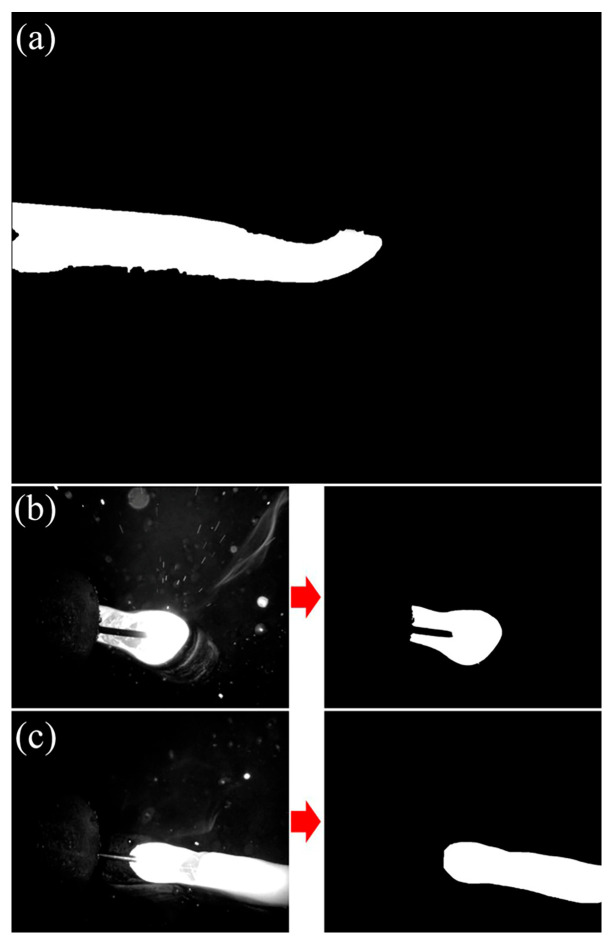
Morphological threshold segmentation results: (**a**) segmented weld bead image after removing background interference; (**b**) real-time molten pool morphology extracted from the same deposition pass; (**c**) corresponding real-time weld bead image showing the solidified bead contour.

**Figure 10:**
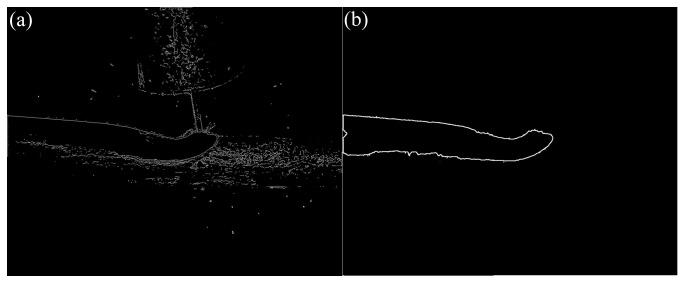
Extraction of weld images using the Sobel edge detection algorithm. (**a**) Detection on the original image; (**b**) Extracted weld contour.

Morphological threshold segmentation operates on binarized images at the pixel level based on structuring elements, with core operations including erosion, dilation, opening, and closing. In this study, the screening criteria for weld morphology were first established based on the binarized weld images as follows: “Area ≥ minimum area, circularity ≥ threshold, and eccentricity ≤ threshold”. Specifically, the minimum area criterion is used to filter out tiny noise points and prevent interfering pixels from being misclassified as targets; circularity measures the area of noise spots that are approximately circular in shape; and the threshold controls the screening sensitivity. The corresponding images after morphological threshold segmentation are shown in Figure 9. Regarding the selection of structuring elements, both elongated and elliptical structuring elements were adopted, with the circular structuring element having a radius of 2 px. The screening parameters were set as follows: minimum pixel area of the weld bead at 500 px^2^, minimum circularity threshold at 0.4, and maximum eccentricity threshold at 0.4. Subsequently, closing and opening operations were sequentially performed to fill internal holes within the weld, connect fragmented weld edges (Figure 9a), and remove isolated noise spots and bright speckles (Figure 9b,c), thereby effectively eliminating background interference and obtaining the morphological threshold-segmented images. Figure 10 presents the weld bead and molten pool images after morphological threshold segmentation.

#### 3.4.2. Weld Edge Contour Extraction

After morphological threshold segmentation of the weld bead images, it is necessary to perform edge feature segmentation and extraction to obtain edge information features of the weld, enabling further analysis of its geometry, dimensions, and dynamic behavior. By comparing the extracted features with actual measurements, the accuracy (mean error and precision) of the dynamic monitoring system in feature size determination can be evaluated, thereby achieving real-time trajectory tracking of the weld. Edge feature extraction also facilitates the assessment of whether the weld morphology is regular and whether defects such as cracks or porosity are present. Currently, commonly employed edge detection algorithms are primarily categorized into first-order and second-order types. First-order edge detection operators are typically represented by the Sobel and Robert algorithms, while second-order operators are mainly represented by the Laplacian and Canny algorithms. In this study, all four aforementioned algorithms were selected for weld contour extraction. Figure 10 presents the results of weld edge contour extraction using the first-order Sobel edge detection algorithm. Although this algorithm enables rapid localization of edge contours, it exhibits insufficient sensitivity to edge responses in the original image (as shown in Figure 10a), resulting in the extraction of numerous interfering points and relatively blurred detected edge contours (as shown in Figure 10b).

**Figure 11:**
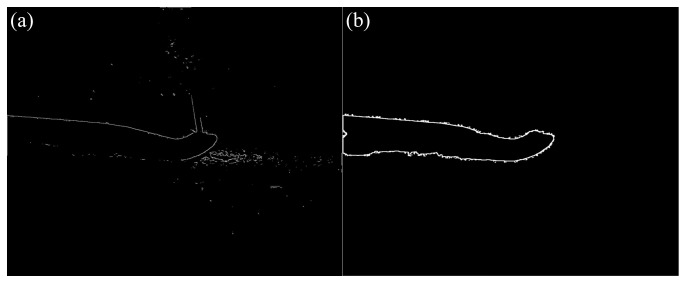
Weld edge contour extraction results by the Robert edge detection algorithm. (**a**) Original image detection; (**b**) Extracted weld contour.

Figure 11 presents the results of weld edge contour extraction using the Robert edge detection algorithm. It can be observed that the detection performance of this algorithm is significantly superior to that of the Sobel edge detection algorithm, with higher localization accuracy of the weld edges. However, its drawback lies in the absence of image smoothing, which results in the loss of partial edge contour information and consequently renders the weld edge contours somewhat blurred.

**Figure 12:**
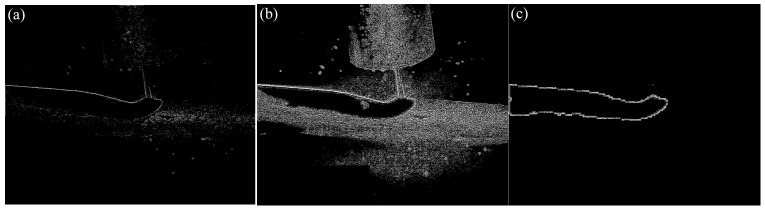
Extraction results of weld edge contours using the second-order Laplacian edge algorithm. (**a**) Threshold is 10; (**b**) Threshold is 1; (**c**) Actual extracted weld contour.

The Laplacian edge detection algorithm, as one of the most classical second-order edge detection operators, offers advantages, including conceptual simplicity, fast computational speed, high localization accuracy, good efficiency, and ease of implementation. Compared with first-order edge detection operators, it exhibits higher sensitivity to edge details. Figure 12 presents the results of weld edge contour extraction using the second-order Laplacian edge detection algorithm. When the threshold sensitivity of the detection algorithm was set to 10 (as shown in Figure 12a), certain detailed features of the weld were automatically disregarded by the algorithm, resulting in incomplete edge detection. When the threshold sensitivity was reduced to 1, the detection range expanded; however, interfering noise points were also misclassified as weld contour edges (as shown in Figure 12b), leading to defects such as loss of weld edge contours and the occurrence of double edges (as shown in Figure 12c). Consequently, the detection results under this parameter setting are also considered unsatisfactory.

**Figure 13:**
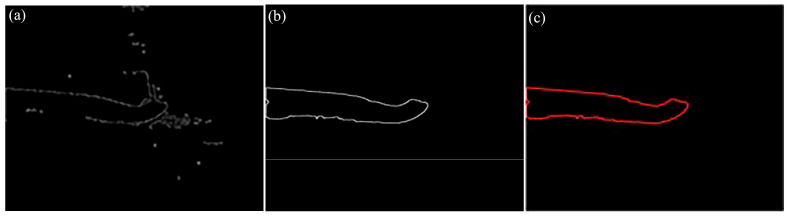
Weld edge contour extraction results obtained by the second-order Canny edge detection algorithm. (**a**) Edge detection; (**b**) Hough transform; (**c**) Linear fitting.

When the Canny edge detection algorithm is employed for weld edge contour extraction, it leverages non-maximum suppression and double-threshold processing mechanisms to ensure single-pixel-width edges with good continuity, thereby accurately capturing the subtle features of irregular weld contours and effectively preserving the integrity of the weld contour. Therefore, the Canny algorithm is considered the optimal choice for weld edge contour extraction in this study. Figure 13a presents the edge contour extraction results of the weld using the Canny algorithm, from which it can be observed that this algorithm preserves the weld edge details to the greatest extent. Furthermore, to enhance the clarity of the weld edge contours, the extracted weld edge contours were further processed using Hough transform for straight-line fitting (as shown in Figure 13b,c). This step eliminates abrupt line segments and smooths the weld contour segments, ultimately yielding a complete weld edge contour image.

#### 3.4.3. Weld Bead and Molten Pool Geometric Morphology

**Figure 14:**
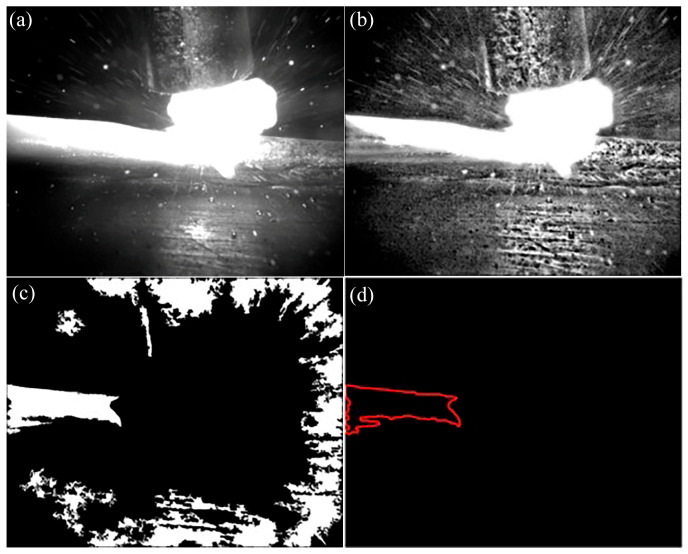
Image processing workflow for liquid metal explosion defects. (**a**) Original image; (**b**) Image enhancement; (**c**) Binarization; (**d**) Edge contour extraction.

The molten pool serves as the “instantaneous core unit” during the forming process, acting as the dynamic carrier of molten metal accumulation, while the weld bead functions as the “solidified fundamental unit,” representing the final morphology after the solidification of a single deposited track. The geometric morphologies of both entities provide intuitive and quantifiable characterizations of the thermo-mechanical-mass transfer equilibrium state during wire arc additive manufacturing. Therefore, it is essential to conduct an in-depth analysis and discussion of their geometric features. However, in actual WAAM processes, the deposition mode is characterized by “short-circuit transfer,” which tends to induce explosive phenomena in the liquid metal (as shown in Figure 14a), resulting in abnormal weld morphologies. During image enhancement (as shown in Figure 14b), although the contrast of the weld morphology is significantly intensified and pixel values converge toward 0 or 255, the trajectories of spatter particles are also manifested as bright line segments. Consequently, during the subsequent grayscale binarization stage (as shown in Figure 14c), substantial loss of the weld feature region occurs, while background interference and spot noise are markedly amplified. This ultimately leads to the loss of weld edge contours, with only partial weld features being detectable (as shown in Figure 14d).

**Figure 15:**
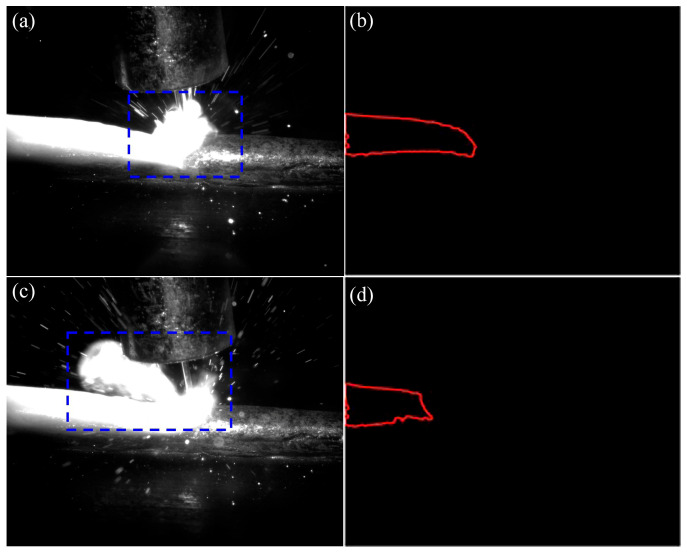
Extracted edge contour images of abnormal weld seams. (**a**) Original image of local spatter; (**b**) Contour extraction of the spatter image; (**c**) Original image of arc tailing; (**d**) Contour extraction of the tailing image.

In addition, various process defects are frequently encountered during actual wire arc additive manufacturing, such as spatter interference caused by excessive welding current (as shown in blue dashed box in Figure 15a) and weld tailing phenomena resulting from arc magnetic blowout (as shown in Figure 15c). The extraction of such abnormal weld images adversely affects the accuracy of geometric morphology detection for both the weld bead and the molten pool. To address this issue, the first five layers of the multi-layer single-pass WAAM process were selected as the specific research subject in this study. The weld formation within this region is relatively stable and can effectively reflect the influence patterns of welding process parameters during the initial stage, demonstrating strong representativeness (Figure 15b,d). During the experiment, five welding video segments within this stage were randomly intercepted. To ensure the timeliness and completeness of image acquisition, the selected videos were decomposed frame by frame at a sampling rate of 30 frames per second. The image data obtained after decomposition were recorded in detail in Table 4 for subsequent unified organization and analysis.

**Figure 16:**
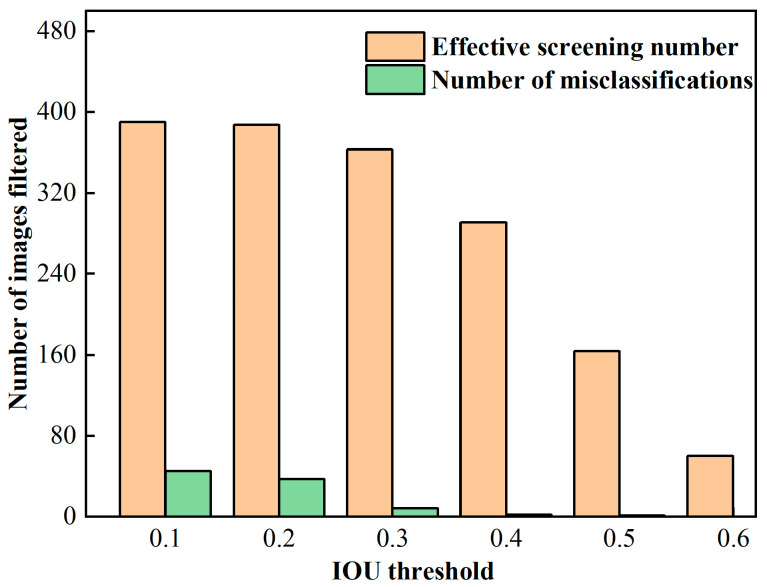
Weld morphology results obtained at different Intersection over Union (IoU) thresholds.

Figure 16 presents the screening results of normal weld morphology under different Intersection over Union (IoU) threshold conditions. It can be observed that as the IoU value increases, both the number of valid screened images and the number of misclassified images decrease. This indicates that a higher IoU threshold corresponds to more stringent image screening criteria, resulting in a lower proportion of defective images being included. However, the overall image detection accuracy for the welding video does not monotonically increase with rising IoU values. On the contrary, as shown in Table 5, an increase in the IoU threshold leads to more refined screening of the original dataset, causing some valid images to be misclassified as interfering images, which consequently reduces the detection accuracy and impairs the monitoring effectiveness for dynamic tracking of the weld bead and molten pool. With comprehensive consideration of both the total sample size and detection accuracy, an IoU threshold of 0.3 was selected as the image screening criterion in this study. Under this threshold, the detection rate of the original dataset exceeds 91%, while the true detection accuracy also remains above 90%, thereby satisfying the requirements for dynamic tracking of the weld bead and molten pool.

## 4. Conclusions

(1) To address the complex interferences encountered during wire arc additive manufacturing, including intense arc light, spatter particles, and welding fume, this study systematically compared the processing effects of mean filtering, Gaussian filtering, median filtering, maximum filtering, and minimum filtering on raw weld bead images. The results indicate that although mean filtering and Gaussian filtering can smooth the grayscale distribution of the images, they fail to effectively eliminate the bright noise spots caused by spatter. Median filtering yields certain improvements but still leaves residual interference. Maximum filtering paradoxically exacerbates the noise effect, whereas minimum filtering effectively removes bright white spots while fully preserving the detailed structure of the weld bead main body, without introducing excessive smoothing or edge blurring. On this basis, adaptive threshold binarization combined with the adapthisteq image enhancement algorithm was further employed to improve both the contrast and contour clarity of the weld region.

(2) Following image preprocessing, the extraction capabilities of four edge detection algorithms—Sobel, Robert, Laplacian, and Canny—were compared for weld contour extraction. The results show that the Sobel operator exhibits low edge sensitivity and generates numerous interfering points; the Robert operator achieves higher localization accuracy but tends to cause edge loss; and although the Laplacian operator offers fast computational speed, it is prone to producing double edges and noise misjudgments at low threshold settings. In contrast, the Canny algorithm, leveraging its non-maximum suppression and double-threshold processing mechanisms, is capable of extracting continuous, single-pixel-width weld edge contours with high integrity. Further straight-line fitting using the Hough transform effectively eliminates abrupt line segments and achieves smoothing of the weld edges. By employing the pixel counting method in conjunction with camera calibration parameters, geometric features, including weld pixel area, molten pool height, and width, were successfully converted into actual physical dimensions, thereby providing technical support for quantitative quality assessment of the fabricated components.

(3) To address the interference phenomena such as spatter, metal explosion, fume occlusion, and arc tailing present in a large number of images obtained from video decomposition, the Intersection over Union (IoU) metric was introduced as the similarity evaluation index between the original images and the standard reference image. Through analysis of the screening performance of 397 original molten pool images under different IoU thresholds, it was found that at IoU = 0.3, the number of valid screened images reached 363, corresponding to a screening rate of 91.4%, with only 6 misclassified images, yielding a misclassification rate of 1.6% and a detection accuracy of 90.0%. Although further increasing the IoU threshold could reduce the misclassification rate, it would lead to the erroneous exclusion of a substantial number of valid images, resulting in a significant decline in detection accuracy. Therefore, IoU = 0.3 was determined as the optimal threshold that balances image quality and detection reliability. This screening strategy significantly enhances the validity of input images in the dynamic monitoring system and effectively prevents non-normal weld morphologies from interfering with feature extraction and analysis results.

(4) In future work, deep learning methods can be further integrated to construct an end-to-end semantic segmentation and defect recognition model for molten pool images, thereby improving robustness against abnormal weld morphologies such as spatter, tailing, and liquid metal explosion. Meanwhile, the image analysis results can be correlated and modeled with process parameters (e.g., current, voltage, wire feed speed, and interpass temperature) to enable online quality prediction and closed-loop control. This would facilitate the transition of wire arc additive manufacturing from “post-deposition inspection” to “intelligent process regulation.”

## Figures and Tables

**Table 1:** Chemical composition of the stainless steel welding wire (wt%).

Elements	C	Si	Mn	S	Cu	P	Cr	Ni	Mo	Fe
ER-316L-1.2	0.02	0.4	1.3	0.01	0.02	0.02	18.7	12.0	2.5	Bal.

**Table 2:** Intrinsic and extrinsic parameters of camera calibration.

Parameters	Values
Camera intrinsic parameter matrix (M)	$\left[\begin{array}{ccc} 5930.7 & 0 & 833.2\\ 0 & 5996.5 & -355.6\\ 0 & 0 & 1 \end{array}\right]$
Camera focal length	$\left[5930.7,5996.5\right]$
Camera principal point	$\left[833.1573,-355.6106\right]$
Camera skew coefficient	0
Camera radial distortion coefficients	$\left[k_{1},k_{2}\right]=\left[0.25,-1.73\right]$
Camera tangential distortion coefficients	$\left[p_{1},p_{2}\right]=\left[-0.0670,-0.0106\right]$

**Table 3:** Parameters used in adaptive thresholding.

Neighborhood Size	Threshold Sensitivity	Minimum Area of Noise (or Minimum Noise Area)
409	0.001	2000 px^2^

**Table 4:** Number of Image Samples.

	Segment 1	Segment 2	Segment 3	Segment 4	Segment 5
Effective welding time (s)	14.00	13.78	13.23	13.07	12.67
Total number of images (frames)	420	413	397	392	380

**Table 5:** Detection accuracy under different intersection over union (IoU) values.

IoU Threshold	0.1	0.2	0.3	0.4	0.5	0.6
Valid count	390	387	363	291	164	60
Screening rate	98.3%	97.5%	91%	73.3%	41.3%	15.1%
Misclassified count	45	37	6	2	1	0
Misclassification rate	11.5%	9.6%	1.6%	0.7%	0.6%	0
Detection accuracy	87.0%	88.1%	90.0%	72.3%	40.7%	15.1%

## Data Availability

All data generated or analyzed during this study are original and were produced by the authors. No third-party data were used. The data are not publicly available but can be obtained from the corresponding author upon reasonable request.
